# Crowdsourcing the Corpasome

**DOI:** 10.1186/1751-0473-8-13

**Published:** 2013-06-21

**Authors:** Manuel Corpas

**Affiliations:** 1 Independent, Cambridge, UK

**Keywords:** *Corpasome*, Facebookome, Shitinome

## Abstract

The suffix -ome conveys “comprehensiveness” in some way. The idea of the Corpasome started half-jokingly, acknowledging the efforts to sequence five members of my family. After the unexpected response from many scientists from around the world, it has become clear how useful this approach could be for understanding the genomic information contained in our personal genomics tests.

## 

Had this article been published earlier, I am sure that *Corpasome* would have been at the top of Jonathan Eisen’s badomics list [1]. According to his article, words with the suffix –ome are meant to convey “comprehensiveness” in some way.

The idea of the Corpasome started half-jokingly as a suggestion from Andrew (Harry) Harrison, acknowledging the efforts to sequence five members of my family. Our efforts started initially with the publications of the 23andMe (https://www.23andme.com) genotype files for the whole Corpas family in 2011 and the creation of myKaryoView [2], a tool specifically designed to visualize personal genomics data from 23andMe and other Direct-to-Consumer (DTC) personal genomics testing companies. After the unexpected response we had from many scientists from around the world, some of them reporting back to us the results of their analyses [3], it became clear how useful this approach could be. At present, croudsourcing is known to be informative for understanding our personal genomes, and consequently, ourselves better. There would be a lot we could share through the Internet, not least our experiences and how our results affected our lives [4].

The Corpasome was born as the accumulation of SNP-derived data for all the family members of the Corpas family who had undergone genotype chip analysis. Although many other pioneering initiatives had been carried out by then such as the Personal Genomes Project (PGP) [5], our approach was original at least in four aspects:

1. All of the experiments were outsourced. We did not actually test the genetic material; most of the analyses were carried out by third party scientists or companies.

2. All of the data pertained to a whole family, as opposed to the individual-based analyses carried out by the PGP. The value of genetic studies having family related individuals is significantly greater as it allows calculation of provenance of traits.

3. All of the data, results and conclusions were made public as soon as they were sent to us. Credit was duly shared or acknowledged as appropriate in any publications or writings derived from the study.

4. We started with no public money whatsoever, it was all a private endeavor. We did this completely independently with private funds and no official support.

It was clear from the start that no single company or DTC provider could or would be able to provide all available knowledge about our personal genomes. Personal genomics tests from DTC companies are mostly designed for an individual anyway, so it was nearly impossible for these companies to answer all or some of our questions. Hence, our experience highlighted the need for open source personal genomics resources tailored to family-specific questions. For instance, given a particular allele, which parent has it been inherited from?

Publishing the Corpasome has not been free from criticism. Some critics mentioned that the informed consent of family members was not truly informed because they are not experts in the field. This comment is somewhat disconcerting, because it does not only assume that I have no knowledge of my family but that they were coerced to take these tests. To some extent, they are right. Their consent is not *fully* informed. But the same is true for any person who has ever taken a personal genomics test. No consent can ever be fully informed, not even the consent of those who call themselves geneticists. This claim is based on the fact that there is always an element of uncertainty in the results: one can never be ready to accept personal genomics results until one knows them, at which point it is too late. Another criticism that we have encountered is that we do this to for the wrong reason, perhaps just to be famous. My answer to that is ‘yes’, we want to widen attention for the project in so far that it allows us to obtain funds to carry on with our experiments. Deep down, however, that is not our ultimate purpose.

You may ask then, what is our ultimate purpose? The *full disclosure* that our ultimate purpose is: for the fun of it. But before you start thinking about how reckless our purpose is or how it can be fun to be predicted to have a high risk of prostate cancer, please let me elaborate a bit more on our meaning of fun. Doing something for fun may have more complex connotations than you would expect. The meaning of fun in this context implies that today it is still a real challenge for a family to analyze their genomes. For ordinary folk, in which the Corpas family can be included, it is very difficult to access this new wealth of knowledge which clinicians and researchers have enjoyed for almost a decade. The difficulty is not only a consequence of the fears that some people associate with publishing our genome data online. It seems that there is a kind of genetic exceptionalism; publishing one’s personal genome is worse than publishing one’s bank account details. Our reaction to this affirmation is, where is the evidence? Isn’t our *Facebookome* more revealing than any genetic information?

Truth be told, there is an element of raising awareness and evangelization to the general public that we embrace here as well. We are lucky enough to live in countries where access to health care is universal and where there is not much to lose even if our insurers were to get hold of these data. Finally, another important element for our Corpasome initiative involves the realization that there is a systemic lack of open source tools and data with which to perform personal genomics analyses. There are very few free tools or public family data available with which to design and develop new analysis tools.

We would like to see this change. We would like to help stimulate the development of a thriving community that provides free tools and models for personal genomics analyses.

We thus released our personal genomic data to the public with absolutely no strings attached to them; you could do everything you like and you would not have to report to us what you find or do with these data and tools. In spite of this, we still would be grateful for any feedback on anything interesting about us. We believe that discoveries beyond our wildest imagination lie just before us. We would rather know them sooner rather than later.

Below are the datasets that have been released as part of the Corpasome initiative. They are available via figshare (http://goo.gl/xsZTF):

• 1 version 2 23andMe genotype set (~0.5 M SNPs)

• 4 version 3 23andMe genotype sets (~1M SNPs each)

• 1 exome

• 1 trio exome

• myKaryoView, a tool for visualization of DTC genotype data

• Figure indicating a deletion inferred from genotype data

• Figure for ISCA analysis for quartet missing grandfather, missing grandmother, mother and aunt.

• A list of 23andMe SNPs for which SNPedia annotations are available.

• Metagenomics data from a fecal sample of one of us (a personal *shitinome*, as we call it).

Following some successful crowdsourcing projects [6,7] any data contributed will be added to figshare and duly acknowledged. We welcome any constructive criticisms to our approach.
